# Dermoscopic Features of Median Rhomboid Glossitis

**DOI:** 10.3390/diagnostics15162047

**Published:** 2025-08-14

**Authors:** Martyna Sławińska, Wojciech Biernat, Beata Zagórska, Michał Sobjanek

**Affiliations:** 1 Department of Dermatology, Venereology and Allergology, Medical University of Gdańsk, 80-214 Gdańsk, Poland; beatazagorska@gumed.edu.pl (B.Z.); michal.sobjanek@gumed.edu.pl (M.S.); 2 Department of Patomorphology, Medical University of Gdańsk, 80-214 Gdańsk, Poland; wojciech.biernat@gumed.edu.pl

**Keywords:** median rhomboid glossitis, dermoscopy, histopathology

## Abstract

We present the case of a 26-year-old, otherwise healthy female patient who attended dermatology outpatient clinic due to a reddish, asymptomatic plaque located on the midline of the tongue. Dermoscopic examination revealed an area of small, atrophic filiform papillae. Based on clinical and dermoscopic features, median rhomboid glossitis (MRG) was suspected. Histopathological analysis confirmed the diagnosis.

**Figure 1:**
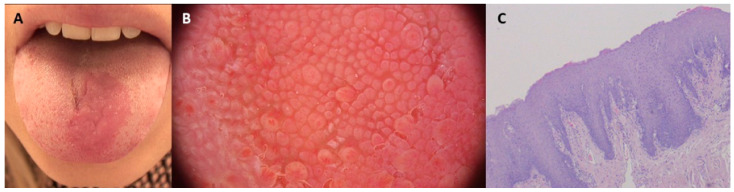
A 26-year-old otherwise-healthy woman presented with an asymptomatic, well-demarcated erythematous area on the dorsum of the tongue (**A**). On dermoscopy, we observed an area in which small, atrophic filiform papillae were observed, different from the normal surrounding tissue (FotoFinder, Vexia Medicam 800 HD; magnification 20×) (**B**). Based on the clinical and dermoscopic presentation, median rhomboid glossitis (MRG) was suspected. Both direct examination and culture were negative. Microscopically, stratified squamous epithelium flattened on the surface with some acanthosis, without visible cellular atypia, and sparse lymphocytic infiltrates in the adjacent lamina propria, were observed with surrounding tissue of normal appearance. Histopathological evaluation confirmed the diagnosis of MRG (**C**). According to data from the literature, the prevalence of MRG ranges from 0.01% to approximately 2.4%, with adult cohorts accounting for between 0.5% and 1%. The prevalence in children is notably lower, at around 0.14% [1,2,3]. MRG typically manifests as a reddish, flat, rhomboid macule or plaque located medially on the dorsum of the tongue. In most cases it is diagnosed based on the clinical presentation. Less commonly it may present as a tumor or in a paramedial location, in which cases it may pose diagnostic difficulties. Differential diagnosis includes atrophic candidiasis, iron deficiency anemia, vitamin B12 deficiency anemia, hemangioma, pyogenic granuloma, Kaposi sarcoma, squamous cell carcinoma, erythroplakia, amyloidosis, or a granular cell tumor [4,5]. The etiology of MRG is not completely understood. Some authors consider this entity to be a subtype of oral candidiasis, while others have suggested an embryological, inflammatory, or autoimmune origin. In the presented case the results of mycological examination and culture were negative (but false negative results cannot be excluded). MRG is associated with good prognosis. In most cases it is asymptomatic, and thus requires no treatment [4]. Coexistence of a corresponding inflammatory area on the palate called candidal infection of the tongue and non-specific inflammation of the palate (CIT-NIP) requires the exclusion of immunodeficiency [5]. Reports in the literature concerning the significance of dermoscopy in the diagnosis of tongue diseases are scant [6,7,8]. The sensitivity and specificity of our observations remain to be elucidated in future studies.

## Data Availability

Not Applicable. No new data were created or analyzed in this study.
